# Electrostatic Contribution of K339 to the Flavin Reduction of *Pseudomonas aeruginosa* PAO1 d‑2-Hydroxyglutarate Dehydrogenase

**DOI:** 10.1021/acsomega.6c09466

**Published:** 2026-10-21

**Authors:** Bilkis Mehrin Moni, Joanna Afokai Quaye, Giovanni Gadda

**Affiliations:** †Departments of Chemistry, ‡Biology, §The Center for Diagnostics and Therapeutics, ∥Molecular Basis of Disease, Georgia State University, Atlanta, Georgia 30302-3965, United States

## Abstract

d-2-Hydroxyglutarate dehydrogenase from *Pseudomonas aeruginosa* PAO1 (*Pa*D2HGDH; EC 1.1.99.39; UniProt ID Q9I6H4) is a Zn^2+^- and FAD-dependent enzyme that catalyzes the oxidation of d-2-hydroxyglutarate to 2-ketoglutarate and has emerged as a potential antibacterial target. A fully conserved positive charge, K339 in *Pa*D2HGDH, is located at the active-site entrance ∼2 Å from the substrate C5 carboxylate, where its positively charged side chain is poised to assist substrate binding via electrostatic interactions. To explore its mechanistic significance, K339 was substituted with methionine, glutamine, or arginine. Rapid-reaction kinetics revealed that K339 substitution weakened d-malate binding (*K*_d_) by ∼5 kJ/mol, reflecting loss or partial attenuation of the electrostatic stabilization provided by the lysine side chain to the substrate C5 carboxylate. The rapid-reaction kinetics pH profile showed that loss of the K339 positive charge shifted the p*K*_a_ of the Zn^2+^–OH­(C2) substrate complex from 8.9 to 10.4, indicating less favorable deprotonation of the substrate C2 hydroxyl in K339 variants. The pH-independent rate constant for flavin reduction, *k*_red_, decreased by up to 80-fold (ΔΔ*G* ∼ 11 kJ/mol) in K339 variants, consistent with the mutation altering the optimal orientation of the substrate C2 hydroxyl group required for flavin reduction. All K339 variants markedly impaired *k*_cat_ and *k*_cat_/*K*_m_, revealing a ∼15 kJ/mol catalytic contribution, while viscosity effects indicated mutation-dependent perturbation of internal isomerization of the enzyme complex during turnover. Overall, K339 plays key electrostatic role in substrate binding and catalysis in *Pa*D2HGDH, with important implications for enzyme engineering and the development of antibacterial inhibitors against *P. aeruginosa*.

## Graphical abstract

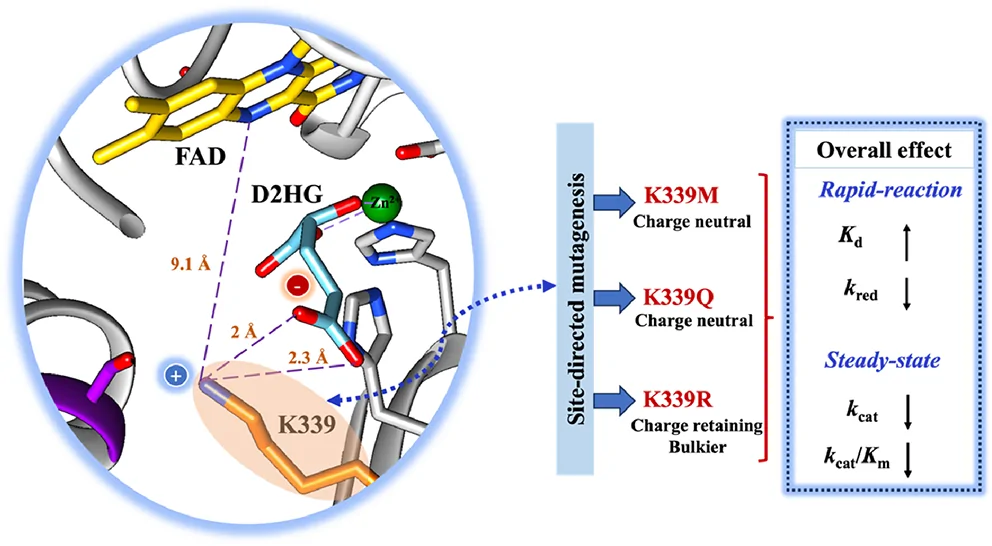

## Introduction

d-2-Hydroxyglutarate dehydrogenase (D2HGDH) has recently received considerable attention due to its physiological substrate, d-2-hydroxyglutarate (D2HG), which is implicated in several cancers and the neurometabolic disorder d-2-hydroxyglutaric aciduria, making the metabolic fate of this oncometabolite clinically relevant.^1–11^ In addition to its central metabolic role, D2HGDH has recently emerged as a recognition element in biosensors for D2HG and related metabolites, further underscoring its biomedical utility.^12–14^ Bioinformatic and biochemical studies have established that D2HGDHs are metalloflavoproteins that use FAD together with a divalent metal ion cofactor to oxidize α-hydroxy acid substrates, with prokaryotic and eukaryotic homologues having diverged into distinct lineages that differ in substrate scope and physiological function.^15^ In general, bacterial D2HGDHs oxidize D2HG and d-malate. In contrast, many eukaryotic D2HGDHs exhibit broader reactivity, including d-lactate, despite the conservation of the overall fold and active-site architecture across the homologues.^15^

Among the D2HGDH homologues, the *Pseudomonas aeruginosa* PAO1 D2HGDH (*Pa*D2HGDH; EC 1.1.99.39; UniProt ID: Q9I6H4) is the most extensively characterized and has become a model system for mechanistic studies.^15–21^ *P. aeruginosa* PAO1 is a multidrug-resistant opportunistic pathogen that causes human nosocomial infections.^22–25^ A previous gene knockout study in *Pseudomonas* species identified D2HGDH as an essential enzyme for bacterial survival by supporting l-serine biosynthesis.^26,27^ In this pathway, *Pa*D2HGDH converts D2HG to 2-ketoglutarate, thereby replenishing cellular 2-ketoglutarate levels required to drive l-serine biosynthesis. Consistent with its proposed central metabolic function, *Pa*D2HGDH has been suggested as a potential therapeutic target against *P. aeruginosa* PAO1.^26^ *Pa*D2HGDH is a Zn^2+^- and FAD-dependent dehydrogenase that catalyzes the oxidation of D2HG to 2-ketoglutarate (Scheme 1) or of d-malate to oxaloacetate.^15–21,28^ In the proposed catalytic mechanism, the active-site metal ion coordinates the C1 carboxylate and C2 hydroxyl groups of the α-hydroxy acid substrate, promotes deprotonation of the C2-OH, and thereby initiates hydride transfer from the substrate α-carbon to the N5 atom of the flavin cofactor (Scheme 1).^15,19,20^ The enzyme displays an optimum pH near 7.4 and requires an active-site proton acceptor with an apparent p*K*_a_ of about 8.3 for efficient flavin reduction.^16,18,19^

**Scheme 1:**
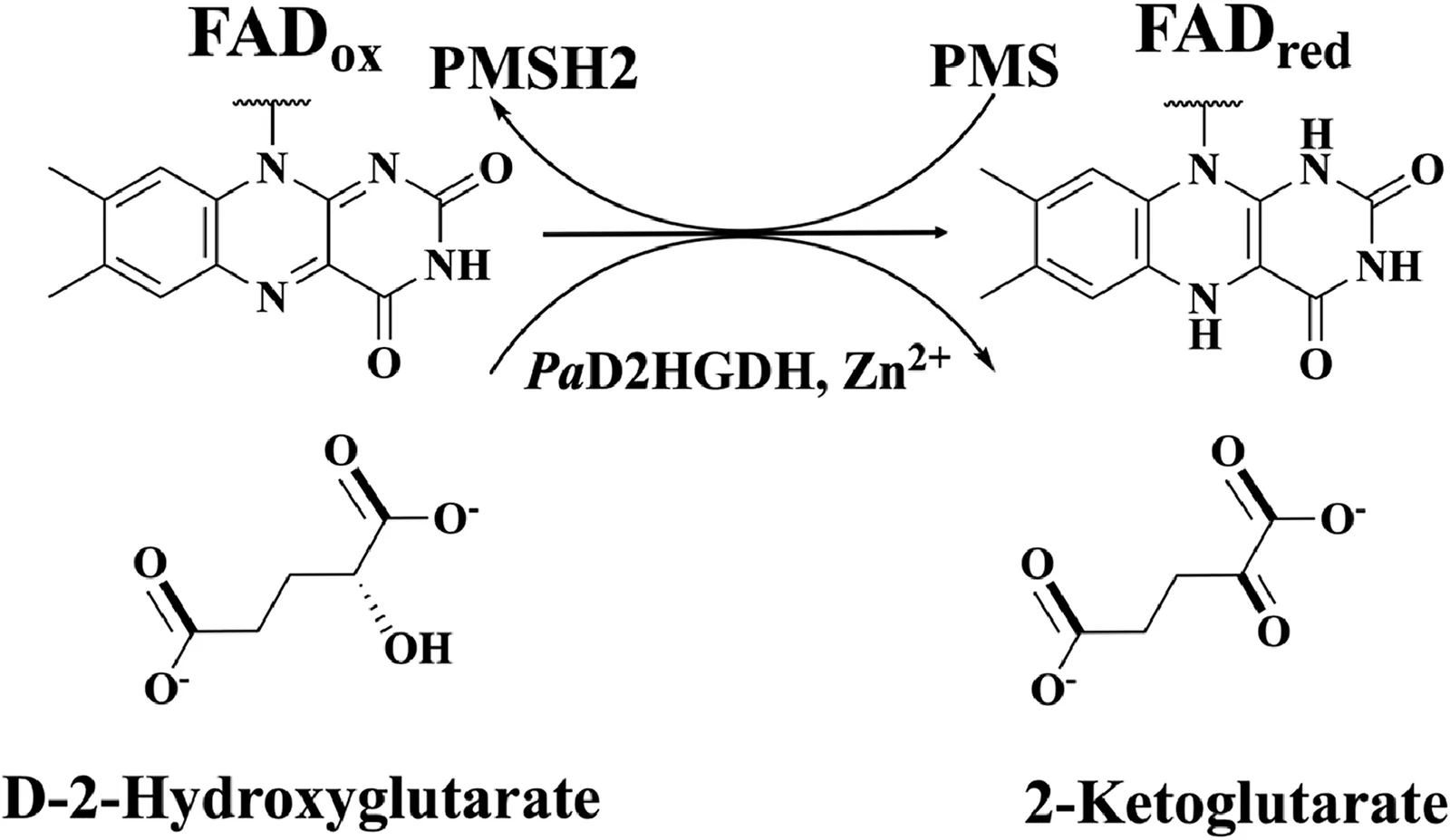
Substrate Oxidation Catalyzed by d-2-Hydroxyglutarate Dehydrogenase from *P. aeruginosa* ^a^

a The physiological electron acceptor for *Pa*D2HGDH has not been established; PMS was used as the artificial electron acceptor in the steady-state assays, with O_2_ consumption resulting from reoxidation of reduced PMS. *Pa*D2HGDH, *P. aeruginosa* d-2-hydroxyglutarate dehydrogenase.

Recent structural and sequence analyses using AlphaFold3 modeling have clarified the conserved features of the *Pa*D2HGDH active site with prokaryotic and eukaryotic D2HGDH homologues.^15^ These models share overall structural features with the published crystal structure of the human D2HGDH homologue and reveal fully conserved FAD-binding and substrate-binding domains, as shown in Figure 1A for *Pa*D2HGDH.^15–18^ The FAD-binding domain exhibits a classical *p*-cresol methyl hydroxylase (PCMH)-type fold with two subdomains and contains a buried pyrophosphate-binding loop (PP-loop) characterized by an X_6_GGXTX_11_ loop-β-strand-loop motif (Figure 1A).^15,29^ Sequence alignment of *Pa*D2HGDH with various D2HGDH homologues identified 36 fully conserved amino acid residues, 18 of which reside in the FAD-binding domain and 18 in the substrate-binding domain.^15^ In the active site, four fully conserved residues form the inner catalytic shell: three of them form a His–Glu–His triad (N–O–N) that coordinates the metal ion together with the flavin O4 atom and the substrate C1 carboxyl and C2 hydroxyl groups, while a fourth histidine (H421 in *Pa*D2HGDH), positioned near the FAD N1/C2 atoms, has been proposed to function as a proton acceptor from the substrate C2-OH during catalysis (Figure 1B).^15,19^ The polar nature of these conserved residues creates a suitable pocket for binding and oxidizing polar α-hydroxy acid substrates via a metal-assisted deprotonation and hydride-transfer mechanism of *Pa*D2HGDH.^15–20^

**Figure 1:**
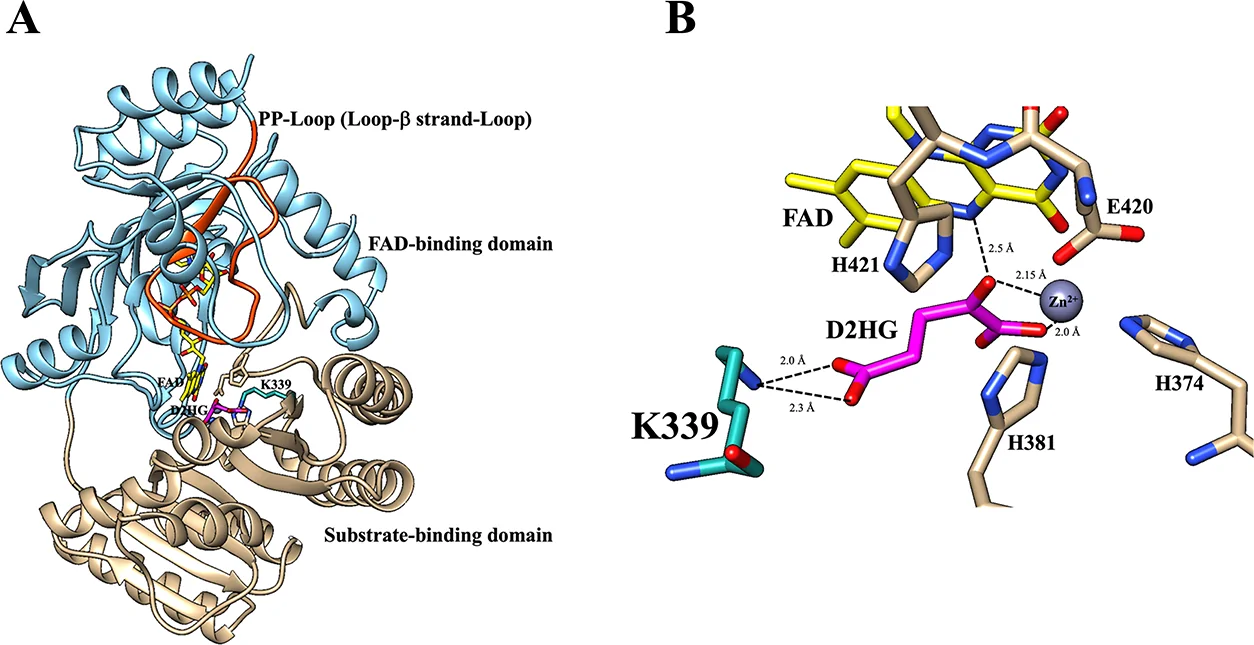
Overall structure and active-site topology of *Pa*D2HGDH with D2HG. (A) Overall structure of *Pa*D2HGDH showing the FAD-binding domain, PP-loop, substrate-binding domain, FAD, Zn^2+^, D2HG, and K339. (B) Enlarged view of the active site showing the spatial relationships among K339, Zn^2+^, FAD, D2HG, and the conserved metal-coordinating residues. Zn^2+^ is modeled as hexacoordinate through the conserved His–Glu–His triad, FAD O4, and the C1 carboxylate and C2 hydroxyl groups of D2HG. Distances between K339 and the substrate and between Zn^2+^ and the substrate are indicated. The model was generated using the Protenix Server from the *Pa*D2HGDH amino acid sequence obtained from the NCBI protein sequence database, together with the CCD code for Zn^2+^ and the SMILES codes for D2HG and FAD.^20,31,32^ FAD is shown in yellow, the FAD-binding domain is in blue, the PP-loop is in orange, the substrate-binding domain and active-site residues are in tan, D2HG is in pink, K339 is in cyan, and Zn^2+^ is in gray. Images were generated using UCSF Chimera software.^30^ *Pa*D2HGDH, *P. aeruginosa* d-2-hydroxyglutarate dehydrogenase.

Besides the inner catalytic shell, structural modeling and multiple sequence alignment of 22 D2HGDH homologues revealed a fully conserved positively charged residue at the entrance to the active site.^15^ In 19 homologues, a lysine is found at this position, whereas an arginine replaces it in the remaining three.^15^ The side chain of the positively charged residue is located ∼3 Å from the distal carboxylate group of bound α-hydroxy acid substrates and is appropriately positioned to interact electrostatically and through hydrogen bonding with the negatively charged substrate tail.^15,20^ Based on its topology and positive charge, the residue has been proposed to contribute to substrate specificity and binding affinity across the D2HGDHs family by stabilizing the terminal carboxylate of the α-hydroxy acid substrate.^15^ In *Pa*D2HGDH, the position corresponds to K339 (Figure 1B), which, together with Zn^2+^, was suggested to assist in substrate capture and orientation, but its mechanistic role had not been investigated experimentally.^15^

A recent study on effector outcomes for a series of *Pa*D2HGDH ligands showed that both the C2 stereochemistry and the charge state of the ligand tail influence whether a given compound behaves as a substrate or an inhibitor.^20^ In that work, d-configured α-hydroxy acids were generally oxidized as substrates, whereas the corresponding l-isomers typically acted as reversible inhibitors.^20^ Variations in chain length and terminal functional group further modulated binding affinity and catalytic competence, consistent with a key contribution from electrostatic interactions between the negatively charged ligand tail and a positively charged residue at the active-site entrance, putatively K339 (Figure 1B). These observations provided strong indirect support for a role of K339 in substrate recognition and in controlling internal equilibria among enzyme–ligand complexes. Nevertheless, that study did not address how K339 influences individual kinetic steps of catalysis or the ionization properties of the active site in *Pa*D2HGDH. Thus, although the enzyme is now well characterized with respect to its metal-dependent mechanism, active-site architecture, and ligand effector behavior, the role of K339 in substrate binding, catalytic turnover, and active-site ionization equilibria remains unclear. In particular, it is not known whether K339 functions primarily to stabilize the Michaelis complex electrostatically, to contribute to the preorganization of the substrate for optimal flavin reduction, to modulate the p*K*_a_ values of catalytic groups within the metal–substrate complex, or to participate in internal isomerization steps of enzyme–substrate (ES) and enzyme–product (EP) complexes.

In this study, we directly probe the mechanistic role of K339 in *Pa*D2HGDH by substituting it with methionine, glutamine, or arginine, thereby systematically perturbing its charge, hydrogen-bonding capacity, and side-chain geometry. We combined UV–visible absorption spectroscopy, steady-state kinetics with D2HG or d-malate, rapid-reaction kinetics, pH-dependent studies of substrate binding and flavin reduction, and kinetic solvent viscosity effects (KSVE) to characterize the wild-type and variant enzymes. By comparing charge-neutral (K339M, K339Q) and charge-retaining (K339R) variant enzymes with the wild-type enzyme, the study delineates the role of K339 in substrate binding, active-site ionization, and internal equilibria in *Pa*D2HGDH, thereby providing new mechanistic insights of relevance to both the broader family of metallo-flavoprotein dehydrogenases and the development of targeted antibacterial strategies against *P. aeruginosa* PAO1.

## Results

### Purification and Spectral Properties of the K339 Variants

Three variants of *P. aeruginosa* d-2-hydroxyglutarate dehydrogenase were engineered using site-directed mutagenesis to substitute the lysine at position 339 with methionine, glutamine, or arginine. The enzymes were independently expressed in the *Escherichia coli* strain Rosetta­(DE3)­pLysS and purified using the same protocol as for the wild-type enzyme.^17–19,21^ The purity was established by SDS-PAGE analysis, which showed a single prominent band corresponding to a molecular weight of ∼51.3 kDa (Figure S3). Approximately 50 mg of each purified variant enzyme was typically recovered from 1.5 L of cell culture. The specific activities of the K339M, K339Q, and K339R enzymes were 8, 7, and 47 U/mg, respectively, with 5 mM d-malate as the substrate at pH 7.4. This value corresponds to a 30-fold decrease with K339M and K339Q and a 5-fold decrease with K339R, respectively, with respect to the specific activity of the wild-type enzyme (with a value of 222 U/mg),^21^ suggesting that K339 is an important residue for enzyme catalysis.

The spectroscopic properties of the K339 variants were analyzed by UV–visible absorption spectroscopy to determine whether the K339 mutation changed the protein microenvironment surrounding the flavin in the enzyme’s active site. The UV–visible absorption spectra of all K339 variants were similar to those of the wild-type enzyme, with absorption maxima near 380 and 450 nm (Figure 2 and Table 1). A small red shift of ∼3 nm was observed near 380 nm, while the molar extinction coefficients differed by less than 5% from those of the wild-type enzyme (Figure 2 and Table 1), indicating that the protein microenvironment surrounding the flavin is not significantly affected by the mutations. The FAD/protein stoichiometry was 0.40 ± 0.01, 0.41 ± 0.02, 0.33 ± 0.01, and 0.46 ± 0.01 for the wild-type, K339M, K339Q, and K339R enzymes, respectively (mean ± SD, *n* = 2 independent protein preparations; Table 1). All kinetic analyses were based on the concentration of FAD-containing enzyme determined from the absorbance of enzyme-bound flavin, thereby minimizing the influence of differences in FAD occupancy on the reported kinetic parameters.

**Figure 2:**
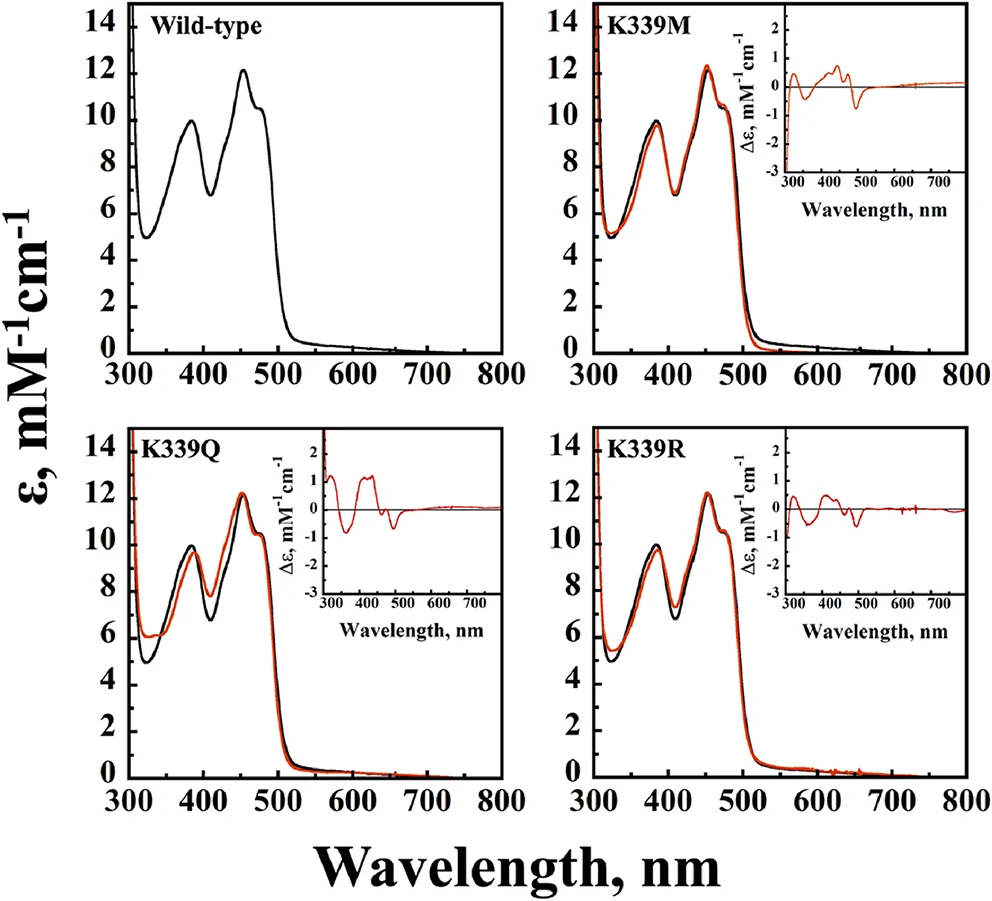
UV–visible absorption spectra of *Pa*D2HGDH wild-type and K339 variants. The UV–visible absorption was recorded in 25 mM NaPO_4_ containing 1 mM ZnCl_2_ and 10% glycerol at pH 7.4 and 25 °C. Insets show the difference between the absorption spectra of the variant enzyme and the wild-type enzyme.

**Table 1:** ^a^UV–Visible Absorption Maxima and FAD/Protein Stoichiometry of *Pa*D2HGDH Wild-Type and K339 Variants

		^b^ε nm, mM^–1^ cm^–1^		
enzymes	^a^λ (nm)	452	∼380	^c^FAD/protein
wild-type	453, 383	12.56	10.04	0.40 ± 0.01
K339M	452, 386	12.30	10.02	0.41 ± 0.02
K339Q	452, 386	12.40	10.13	0.33 ± 0.01
K339R	452, 386	12.24	10.18	0.46 ± 0.01

a The UV–visible absorption spectra were measured in 25 mM NaPO_4_ containing 1 mM ZnCl_2_ and 10% glycerol at pH 7.4 and 25 °C.

b Molar extinction coefficient.

c The FAD-to-protein ratio was determined using the Bradford assay.^33^

### Steady-State Kinetics with K339 Variants

The steady-state kinetics of the K339 variants were investigated to examine how the spatial location of a positive charge at residue 339 in the enzyme’s active site may affect catalysis. The steady-state kinetic parameters were examined at pH 7.4 and 25 °C by measuring the initial rate of oxygen consumption at varying concentrations of the physiological substrate D2HG or the alternative substrate d-malate, with a fixed saturating concentration of 2 mM PMS in 25 mM NaPO_4_ (Tables 2 and 3). Oxygen consumption indicates the reoxidation of reduced PMS, as *Pa*D2HGDH does not react directly with molecular oxygen. Activity measurements at 1, 2, and 3 mM PMS with 60 mM d-malate gave comparable rates at 2 and 3 mM PMS; therefore, 2 mM PMS was used as an operationally saturating concentration under these assay conditions. As illustrated in Tables 2 and 3, substitution of K339 with methionine resulted in a 750-fold decrease in the *k*_cat_ value and a 290-fold decrease in the *k*_cat_/*K*_m_ value with D2HG and a 210-fold decrease in the *k*_cat_ value and a 100-fold decrease in the *k*_cat_/*K*_m_ value with d-malate. Additionally, substitution of K339 with glutamine resulted in a 330-fold decrease in the *k*_cat_ value and a 100-fold decrease in the *k*_cat_/*K*_m_ value with D2HG, and a 220-fold decrease in the *k*_cat_ value and a 140-fold decrease in the *k*_cat_/*K*_m_ value with d-malate (Tables 2 and 3). For comparison, the K339R variant showed a smaller decrease of ∼15-fold in both *k*_cat_ and *k*_cat_/*K*_m_ with D2HG, and ∼40-fold in *k*_cat_ and ∼10-fold in *k*_cat_/*K*_m_ with d-malate (Tables 2 and 3). These data suggest that K339 is crucial for efficient enzyme catalysis. Although the *K*_m_ values changed only modestly (Tables 2 and 3), *K*_m_ is a composite steady-state parameter that can be influenced by multiple microscopic steps, including flavin reduction, product release, and internal isomerization, and should not be interpreted as a direct measure of substrate-binding affinity.

**Table 2:** ^a^Steady-State Kinetics of *Pa*D2HGDH Wild-Type and K339 Variants with Varying D2HG Concentrations and Fixed Saturating PMS

enzymes	*k*_cat_, s^–1^	*K*_m_, mM	*k*_cat_/*K*_m_, M^–1^ s^–1^
wild-type	30 ± 1 ^18^	0.15 ± 0.01 ^18^	205,000 ± 10,000 ^18^
K339M	0.04 ± 0.01	0.06 ± 0.01	700 ± 40
K339Q	0.09 ± 0.01	0.05 ± 0.01	2000 ± 150
K339R	1.70 ± 0.05	0.10 ± 0.01	17,000 ± 1500

a The kinetic parameters of variant enzymes were determined with 2 mM PMS in 25 mM NaPO_4_, pH 7.4, at 25 °C. The variant enzyme K339M, K339Q, and K339R concentrations were 4.6, 2.4, and 0.7 μM, respectively. Standard errors were from individual fits of the kinetic data. The wild-type kinetic parameters were from a previously published paper.^18^ Wild-type kinetic parameters adapted in part from Quaye, J. A.; Gadda, G. The *Pseudomonas aeruginosa* PAO1 Metalloflavoprotein d-2-Hydroxyglutarate Dehydrogenase Requires Zn^2+^ for Substrate Orientation and Activation. *J. Biol. Chem.* 2023, 299 (3), 103008. Copyright 2023 The Authors. Published by Elsevier Inc. on behalf of the American Society for Biochemistry and Molecular Biology under a CC BY 4.0 license.

**Table 3:** ^a^Steady-State Kinetics of *Pa*D2HGDH Wild-Type and K339 Variants with Varying d-Malate Concentrations and Fixed Saturating PMS

enzymes	*k*_cat_, s^–1^	*K*_m_, mM	*k*_cat_/*K*_m_, M^–1^ s^–1^
wild-type	38 ± 1 ^18^	3.8 ± 0.7 ^18^	10,000 ± 1000 ^18^
K339M	0.18 ± 0.01	1.6 ± 0.2	100 ± 10
K339Q	0.17 ± 0.01	2.6 ± 0.4	70 ± 10
K339R	1.02 ± 0.02	1.0 ± 0.1	1000 ± 100

a The kinetic parameters of variant enzymes were determined with 2 mM PMS in 25 mM NaPO_4_, pH 7.4, at 25 °C. The variant enzyme K339M, K339Q, and K339R concentrations were 4.6, 2.4, and 0.7 μM, respectively. Standard errors were from individual fits of the kinetic data. The wild-type kinetic parameters were from a previously published paper.^18^ Wild-type kinetic parameters adapted in part from Quaye, J. A.; Gadda, G. The *Pseudomonas aeruginosa* PAO1 Metalloflavoprotein d-2-Hydroxyglutarate Dehydrogenase Requires Zn^2+^ for Substrate Orientation and Activation. *J. Biol. Chem.* 2023, 299 (3), 103008. Copyright 2023 The Authors. Published by Elsevier Inc. on behalf of the American Society for Biochemistry and Molecular Biology under a CC BY 4.0 license.

### Reductive Half-Reaction of K339 Variants

As the initial step toward the mechanistic characterization of d-malate oxidation by the K339 variants, the reductive half-reaction was examined by anaerobic mixing of the K339 variants with d-malate by observing the rates of decrease in absorbance at 450 nm as a function of the concentration of the substrate in a stopped-flow spectrophotometer at pH 7.4 (Figures 3, S1, and S2) and pH 9.5. Anaerobic conditions were used to isolate substrate-driven FAD reduction and prevent competing reoxidation of the reduced flavin. pH 7.4 was chosen because earlier results with the wild-type enzyme showed that the kinetic parameter *K*_d_ was pH-independent around pH 7.4.^19^ In contrast, the high pH was chosen to probe the rate constant for flavin reduction (*k*_red_) to avoid artificial contributions from pH effects, since previous results with the wild-type enzyme showed that the kinetic parameter *k*_red_ is pH-independent at high pH.^19^ The K339 variant’s enzyme-bound flavin was reduced to the hydroquinone state in a biphasic pattern similar to the previously published wild-type enzyme (Figures 3, S1, and S2).^18^ The fast phase accounted for more than 90% of the total absorbance change at 450 nm and was attributed to flavin reduction.^18^ The slow phase accounted for ∼10% of the total absorbance change, with a substrate-concentration-independent *k*_obs_ value of ∼0.2 s^–1^, likely arising from some nonfunctional enzyme generated during the anaerobic sample preparation.^18^ The observed rates of flavin reduction of the K339 variants were hyperbolically dependent on the concentration of d-malate, yielding 75-fold, 18-fold, and 2-fold lower *k*_red_ values of K339Q, K339R, and K339 M variants, respectively, compared to the wild-type enzyme at pH 9.5 (Table 5). The *K*_d_ values of K339 M were 10-fold higher, and those of K339Q and K339R were ∼3-fold higher than the wild-type enzyme at pH 7.4 (Table 4), suggesting that the positive charge at position 339 is important for the binding of the α-hydroxy acid substrate in *Pa*D2HGDH. The chemical step of flavin reduction in the reverse direction, *k*_rev_ of K339M and K339Q, was not significantly different from zero at both pH 7.4 and 9.5, while the *k*_rev_ values of the K339R and wild-type enzymes were comparable with small but not negligible values (Tables 4 and 5). The detectable *k*_rev_ for wild-type and K339R variants indicates partial reversibility of the reductive half-reaction. In contrast, the absence of measurable *k*_rev_ for K339M and K339Q suggests that the reverse step is strongly disfavored under the experimental conditions.

**Figure 3:**
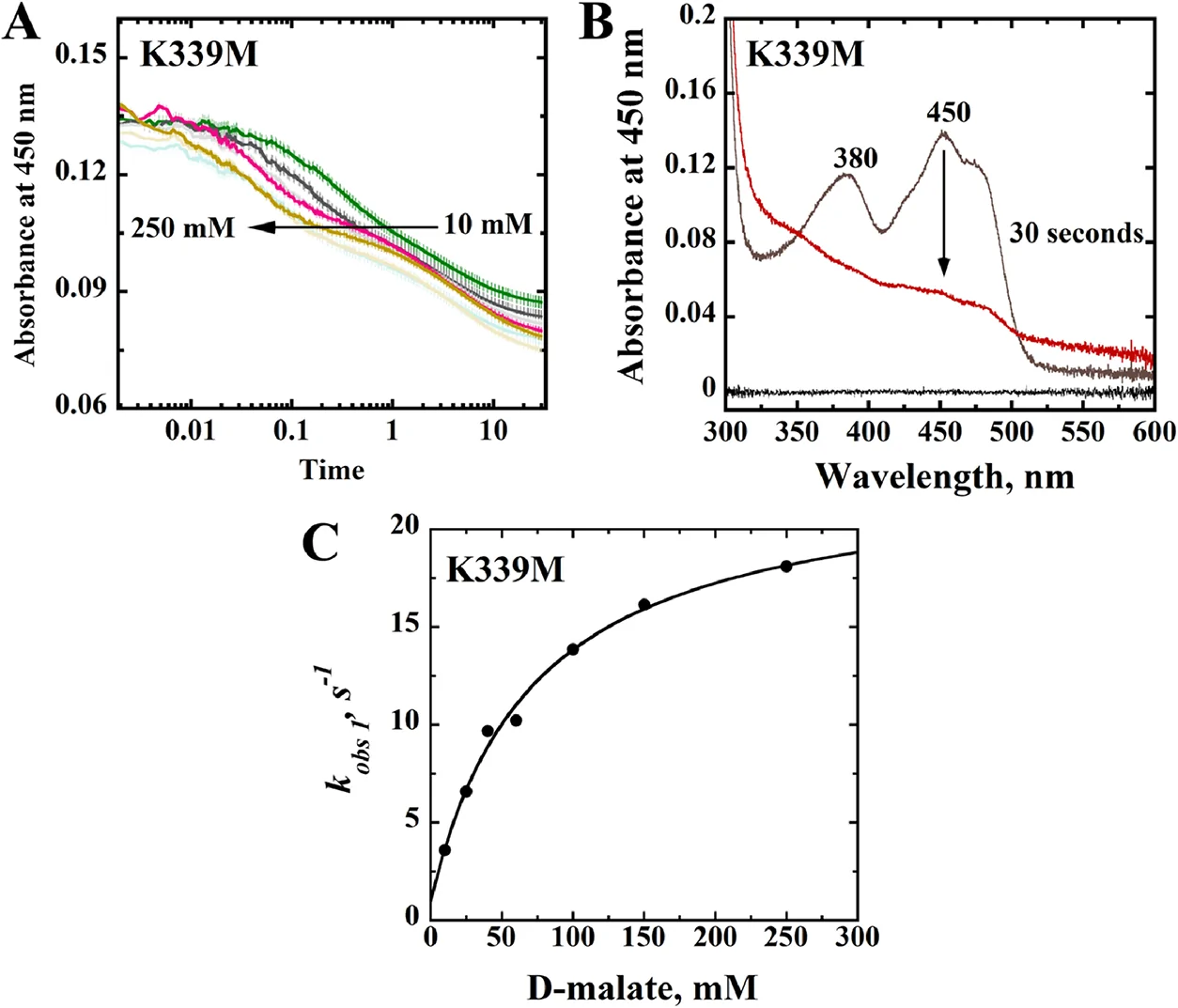
Anaerobic reduction of the K339M variant with d-malate at pH 7.4. (A) Stopped-flow traces of K339M at 450 nm with varying concentrations of d-malate (10–250 mM), fit to eq 3. Note the log time scale. For clarity, one of every ten experimental points is shown (vertical lines). The instrument dead time is 2.2 ms. (B) Absorption spectra of K339M showing the fully oxidized flavin before reduction (gray trace) and fully reduced flavin after reduction with 100 mM d-malate (red trace). (C) Concentration dependence of the observed rate constant (*k*_obs1_) for flavin reduction of K339M with d-malate, fit to eq 4. The experiment was carried out in 25 mM NaPO_4_ using an SF-61DX2 Hi-Tech KinetAsyst high-performance stopped-flow spectrophotometer, thermostated at 25 °C and equipped with a photomultiplier tube detector under anaerobic conditions.

**Table 4:** ^a^Reductive Half-Reaction of *Pa*D2HGDH Wild-Type and K339 Variants with Varying d-Malate Concentrations at pH 7.4

enzymes	*k*_red_, s^–1^	*K*_d_, mM	*k*_rev_, s^–1^
wild-type	68 ± 2 ^18^	8 ± 1 ^18^	4 ± 2 ^18^
K339M	22 ± 1	70 ± 20	–
K339Q	1.2 ± 0.1	20 ± 8	–
K339R	3.4 ± 0.2	26 ± 9	2 ± 0.2

a The rapid reaction kinetic parameters were determined with substrate d-malate in 25 mM NaPO_4_, pH 7.4, at 25 °C. Standard errors were from individual fits of the kinetic data. Symbol “–” indicates close to zero. The wild-type kinetic parameters were from a previously published paper.^18^ Wild-type kinetic parameters adapted in part from Quaye, J. A.; Gadda, G. The *Pseudomonas aeruginosa* PAO1 Metalloflavoprotein d-2-Hydroxyglutarate Dehydrogenase Requires Zn^2+^ for Substrate Orientation and Activation. *J. Biol. Chem.* 2023, 299 (3), 103008. Copyright 2023 The Authors. Published by Elsevier Inc. on behalf of the American Society for Biochemistry and Molecular Biology under a CC BY 4.0 license.

**Table 5:** ^a^Reductive Half-Reaction of *Pa*D2HGDH Wild-Type and K339 Variants with Varying d-Malate Concentrations at pH 9.5

enzymes	*k*_red_, s^–1^	*K*_d_, mM	*k*_rev_, s^–1^
wild-type	570 ± 70 ^19^	20 ± 6 ^19^	–
K339M	225 ± 20	100 ± 20	–
K339Q	7.4 ± 0.1	8.5 ± 0.5	
K339R	32 ± 2	25 ± 6	15 ± 2

a The rapid-reaction kinetic parameters were determined with substrate d-malate in 25 mM Good’s buffer (0.1 M ACES, 0.052 M Tris, and 0.052 M ethanolamine) supplemented with 1 mM ZnCl_2_, pH 9.5, at 25 °C. Standard errors are from individual fits of the kinetic data. Symbol “–” indicates close to zero. The wild-type kinetic parameters were from a previously published paper.^19^ Wild-type data adapted in part from Quaye, J. A.; Gadda, G. Metal-Triggered FAD Reduction in d-2-Hydroxyglutarate Dehydrogenase from *Pseudomonas aeruginosa* PAO1. *ACS Bio Med Chem Au* 2025, 5 (1), 204–214. Copyright 2024 The Authors. Published by the American Chemical Society under a CC BY 4.0 license.

### pH Effect on *k*_red_ and *K*_d_ in the K339M Variant

To investigate the role of the positive charge of K339 in the active-site ionization environment of *Pa*D2HGDH, the anaerobic reductive half-reaction of the K339M variant was carried out in buffered solutions from pH 7.0 to pH 10.5. Among the three enzyme variants, K339M was selected to more clearly assess how the removal of the positive charge and the loss of hydrophobic-binding ability affect the active-site ionization environment of *Pa*D2HGDH. The *k*_red_ and *K*_d_ values were determined between pH values of 7.0 and 10.5 (Figure 4). The wild-type pH profile data were extrapolated from a previously published paper.^19^ The *k*_red_ value increased with increasing pH, reaching plateaus at high pH, as previously established for the wild-type enzyme (Figure 4A and Table S1).^19^ The *k*_red_ was pH-independent above pH 9.0, with a p*K*_a_ of 8.2 ± 0.08. The pH-independent limiting value at high pH was 235 ± 20 s^–1^ for the K339M variant, which was 2.4-fold lower than that of the wild-type enzyme (570 ± 70 s^–1^; Table 6).^19^ The *K*_d_ value increased with increasing pH for both wild-type and variant enzymes (Figure 4B and Table S1).^19^ The *K*_d_ was pH-independent below pH 9.5 in the K339M variant and pH 8.5 for the wild-type enzyme, with apparent p*K*_a_ values of 10.4 ± 0.1 and 8.9 ± 0.1 for the two enzymes, respectively (Table 6).^19^ The p*K*_a_ is reported as an apparent value because the upper plateau was only partially reached within the experimentally accessible pH range (Figure 4). The pH-independent-limiting *K*_d_ value was 75 ± 5 mM for the K339M variant and 3 ± 0.3 mM for the wild-type enzyme (Table 6).

**Figure 4:**
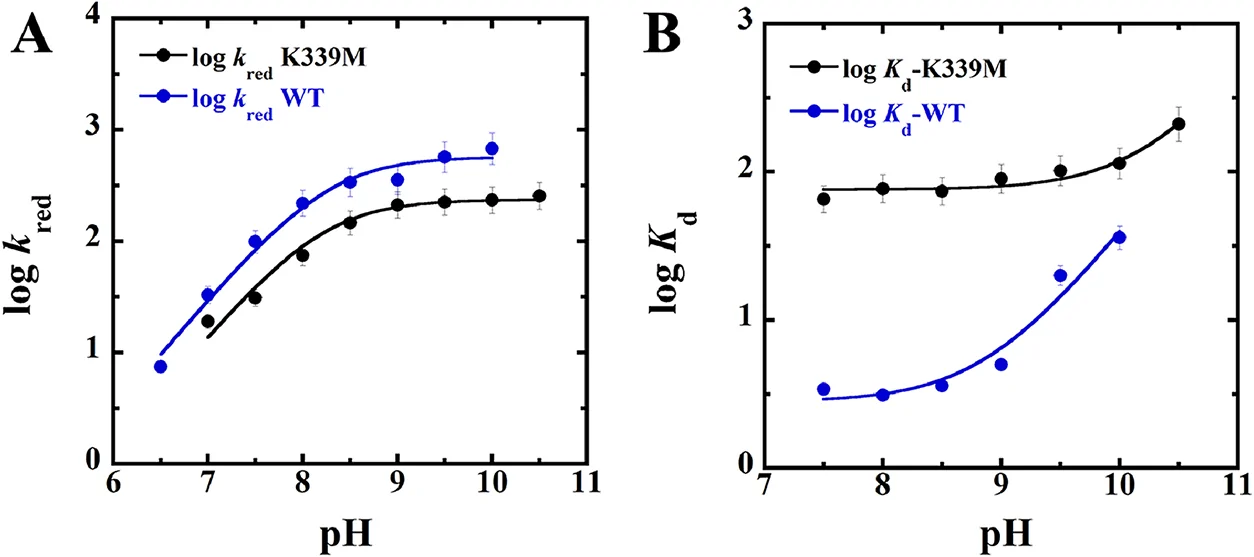
Effect of pH on the reductive half-reaction of *Pa*D2HGDH wild-type and K339M with d-malate between pH 7.0 and 10.5. (A) pH profile of *k*_red_. (B) pH profile of *K*_d_. Data were fit to eqs 7 and 8. The wild-type pH profile data were extrapolated from a previously published paper.^19^ Wild-type data adapted in part from Quaye, J. A.; Gadda, G. Metal-Triggered FAD Reduction in d-2-Hydroxyglutarate Dehydrogenase from *Pseudomonas aeruginosa* PAO1. *ACS Bio Med Chem Au* 2025, 5 (1), 204–214. Copyright 2024 The Authors. Published by the American Chemical Society under a CC BY 4.0 license.

**Table 6:** ^a^Comparison of the Reductive Half-Reaction pH Profile Parameters of *Pa*D2HGDH Wild-Type and K339M with d-Malate

	*k*_red_ pH profile		*K*_d_ pH profile	
enzymes	*k*_red_(lim), s^–1^	p*K*_a_	*K*_d_(lim), mM	p*K*_a_
wild-type	570 ± 70 ^19^	8.3 ± 0.08 ^19^	3 ± 0.3 ^19^	8.9 ± 0.1 ^19^
K339M	235 ± 20	8.2 ± 0.08	75 ± 5	10.4 ± 0.1

a Values were extrapolated from pH profile plots obtained by fitting the kinetic data of the log *k*_red_ or *K*_d_ parameters as a function of pH using eqs 7 and 8, upon K339M variant reduction with d-malate as a substrate between pH 7.0 and 10.5 under anaerobic conditions. Standard errors were from individual fits of the kinetic data. The wild-type pH profile data were extrapolated from a previously published paper.^19^ Wild-type data adapted in part from Quaye, J. A.; Gadda, G. Metal-Triggered FAD Reduction in d-2-Hydroxyglutarate Dehydrogenase from *Pseudomonas aeruginosa* PAO1. *ACS Bio Med Chem Au* 2025, 5 (1), 204–214. Copyright 2024 The Authors. Published by the American Chemical Society under a CC BY 4.0 license.

### Solvent Viscosity Effect on *k*_cat_ and *k*_cat_/*K*_m_ in K339 Variants

The effects of solvent viscosity on *k*_cat_ and *k*_cat_/*K*_m_ using d-malate as the substrate were examined in K339 variants to investigate internal isomerization during the reductive half-reaction. Previous kinetic solvent viscosity effect (KSVE) and pL-profile studies of the wild-type enzyme suggested that an internal isomerization of the enzyme–substrate (ES) complex occurs before flavin reduction.^19,28^ Initial rates of reaction of the K339 variants were measured at varying concentrations of d-malate in 25 mM NaPO_4_, pH 7.4, at 25 °C in the presence of increasing amounts of glucose or glycerol as viscosigens. As shown in Figures 5 and 6, the *k*_cat_ and *k*_cat_/*K*_m_ values of the K339Q variant, as well as the *k*_cat_ value of the K339M variant, increased with increasing the viscosity of the solvent. A plot of normalized kinetic parameters versus relative solvent viscosity showed an inverse hyperbolic relationship, with similar trends across viscosigen types (Figures 5 and 6). These data are consistent with internal isomerization in the reductive half-reaction of the enzyme.^34,35^ In contrast, the plot of normalized *k*_cat_/*K*_m_ for K339M versus relative solvent viscosity yielded slopes of 0.05 with glucose and 0.23 with glycerol (Table 7). The smaller slope with glucose than with glycerol likely reflects viscosity-specific effects due to their different molecular sizes and chemical properties; however, both slopes indicate only a limited viscosity dependence of *k*_cat_/*K*_m_ for K339M. However, for the K339R variant, slopes of 0.03 and ∼0.07 were determined in a plot of normalized *k*_cat_ and *k*_cat_/*K*_m_, respectively, versus relative viscosity of the solvent (Figures 5 and 6 and Table 7), consistent with the rate of product release and the kinetic step of substrate binding minimally limiting the overall turnover of the enzyme.^36^

**Figure 5:**
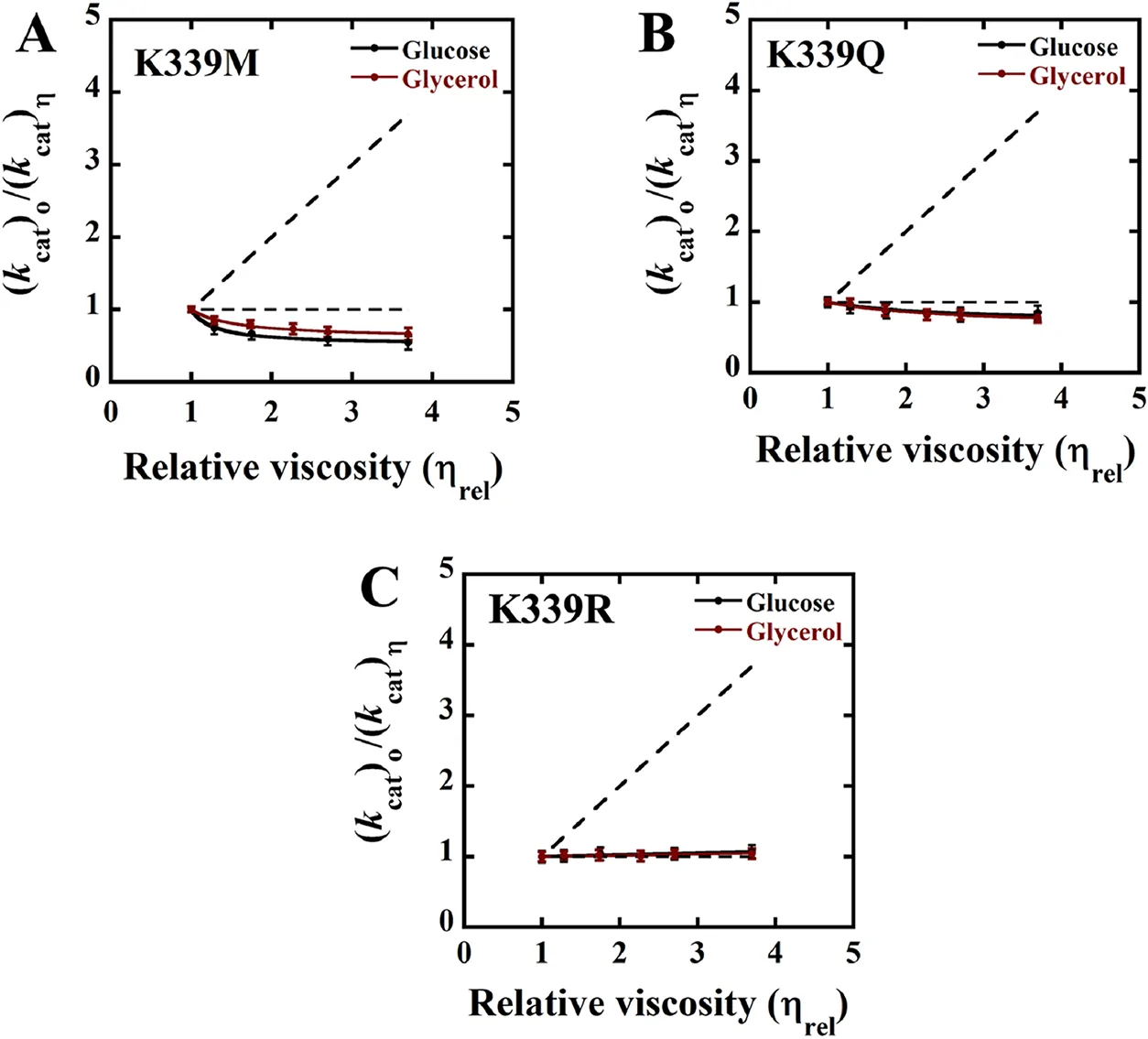
Effect of solvent viscosity on the steady-state kinetic parameter *k*_cat_ for *Pa*D2HGDH wild-type and K339 variants. Panel (A) shows the normalized *k*_cat_ values with d-malate as a function of relative solvent viscosity for the K339 M variant. Panel (B) shows the normalized *k*_cat_ values with d-malate as a function of relative solvent viscosity for the K339Q variant. Panel (C) shows the normalized *k*_cat_ values with d-malate as a function of relative solvent viscosity for the K339R variant. The dashed lines with a slope of 1 indicate the expected results for a fully diffusion-limited reaction. The enzymes activity assays were carried out using a Clark-type oxygen electrode in 25 mM NaPO_4_, 2 mM PMS, pH 7.4 thermostated at 25 °C, containing varying amounts of glycerol: red (0–40%, m/m, η = 1.0–3.5 cP) and glucose: black (0–34%, m/m, η_rel_ = 1.0–3.6 cP). The solid lines show the fit of the data to eqs 5 or 6 for linear or hyperbolic dependencies, respectively.

**Figure 6:**
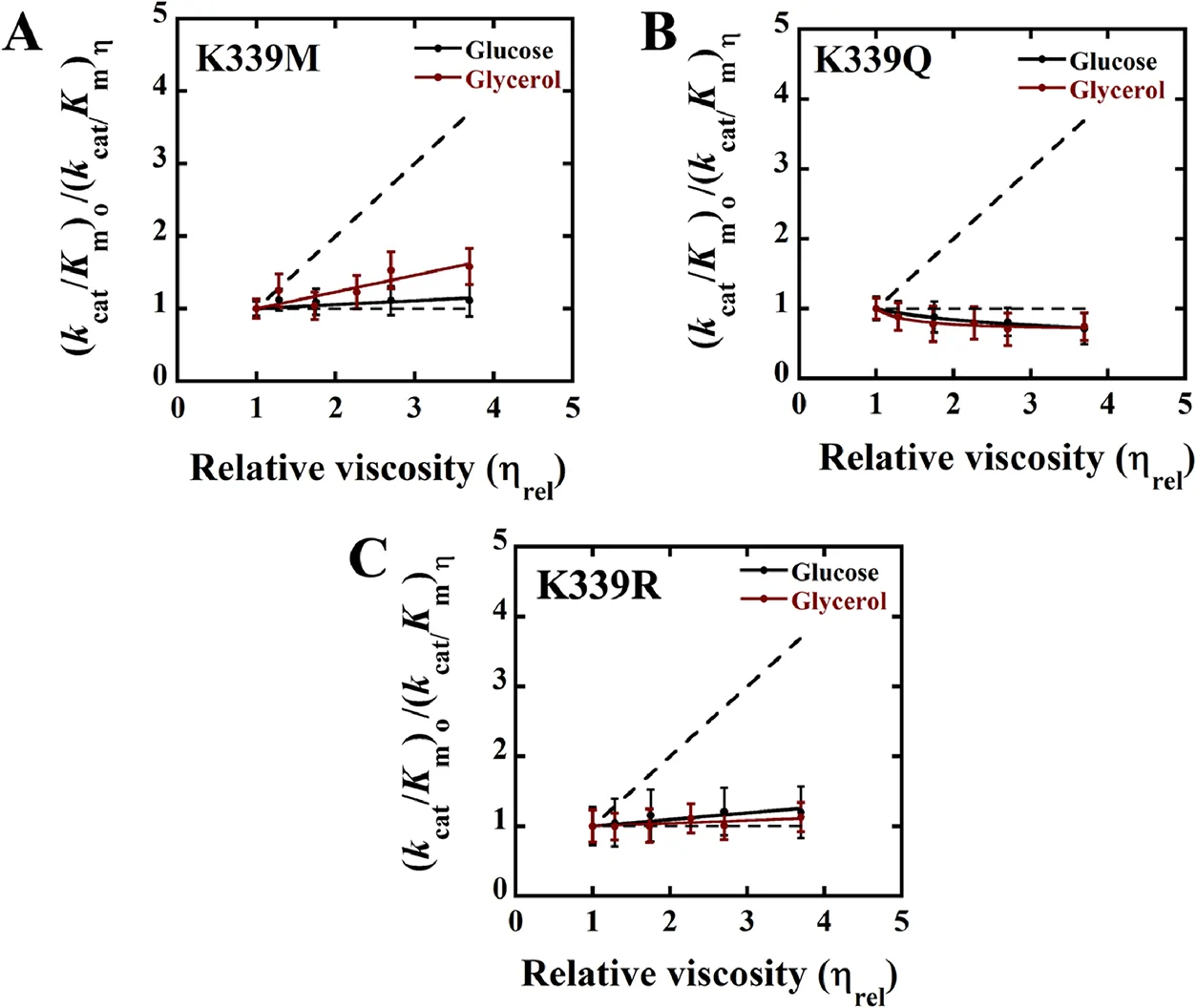
Effect of solvent viscosity on the steady-state kinetic parameter *k*_cat_/*K*_m_ for *Pa*D2HGDH wild-type and K339 variants. (A) Normalized *k*_cat_/*K*_m_ values with d-malate as a function of relative solvent viscosity for the K339M variant. (B) Normalized *k*_cat_/*K*_m_ values with d-malate as a function of relative solvent viscosity for the K339Q variant. (C) Normalized *k*_cat_/*K*_m_ values with d-malate as a function of relative solvent viscosity for the K339R variant. The dashed lines with a slope of 1 indicate the expected results for a fully diffusion-limited reaction. The enzymes activity assays were carried out using a Clark-type oxygen electrode in 25 mM NaPO_4_ and 2 mM PMS, pH 7.4, thermostated at 25 °C, containing varying amounts of glycerol (red, 0–40%, m/m, η = 1.0–3.5 cP) and glucose (black, 0–34%, m/m, η_rel_ = 1.0–3.6 cP). The solid lines report a fit of the data to eqs 5 or 6 for either linear or hyperbolic dependency, respectively.

**Table 7:** ^a^Kinetic Solvent Viscosity Effects on the Steady-State Kinetic Parameters of *Pa*D2HGDH Wild-Type and K339 Variants

	*k*_cat_		*k*_cat_/*K*_m_	
enzymes	glucose	glycerol	glucose	glycerol
wild-type	0.52 ± 0.04 ^18^	0.32 ± 0.02 ^18^	0.34 ± 0.04 ^18^	0.47 ± 0.05 ^18^
K339M	—	—	0.05 ± 0.02	0.23 ± 0.03
K339Q	—	—	—	—
K339R	0.03 ± 0.003	0.03 ± 0.003	0.09 ± 0.02	0.04 ± 0.01

a The enzyme activity assays were carried out using a Clark-type oxygen electrode in 25 mM NaPO_4_, 2 mM PMS, pH 7.4, thermostated at 25 °C, containing varying amounts of glycerol (0–40%, m/m, η_rel_ = 1.0–3.5 cP) or glucose as a viscogen (0–34%, m/m, η_rel_ = 1.0–3.6 cP). The slopes from the viscosity effects were extrapolated after fitting the data to eq 6. —, not applicable, as the KSVE exhibited an inverse hyperbolic dependence rather than a linear relationship; therefore, a linear slope was not determined. Standard errors were from individual fits of the kinetic data. The wild-type kinetic viscosity parameters were from a previously published paper.^18^ Wild-type kinetic parameters adapted in part from Quaye, J. A.; Gadda, G. The *Pseudomonas aeruginosa* PAO1 Metalloflavoprotein d-2-Hydroxyglutarate Dehydrogenase Requires Zn^2+^ for Substrate Orientation and Activation. *J. Biol. Chem.* 2023, 299 (3), 103008. Copyright 2023 The Authors. Published by Elsevier Inc. on behalf of the American Society for Biochemistry and Molecular Biology under a CC BY 4.0 license.

## Discussion

*P. aeruginosa* d-2-hydroxyglutarate dehydrogenase is a Zn^2+^- and FAD-dependent enzyme that catalyzes the oxidation of D2HG to 2-ketoglutarate and d-malate to oxaloacetate. In the proposed catalytic mechanism, the active-site metal ion coordinates the C1 carboxylate and C2 hydroxyl groups of the α-hydroxy acid substrate, promotes deprotonation of the C2-OH, and thereby initiates hydride transfer from the substrate α-carbon to the N5 atom of the flavin cofactor.^15,19,20,28^ In this study, a fully conserved positively charged residue, K339, in the active-site entrance of *Pa*D2HGDH was substituted with methionine, arginine, or glutamine to establish its role in the catalytic cycle of the enzyme. Mutation of K339 decreased the binding affinity for d-malate, consistent with loss of the electrostatic interaction with the substrate C5 carboxylate. Mutation at this position also altered the optimal orientation of the substrate C2 hydroxyl group for flavin reduction. In addition, substitution at position 339 changed the ionization environment of the active site of the enzyme by increasing the p*K*_a_ of the Zn^2+^–OH­(C2) substrate complex. The mutagenesis and mechanistic investigation reported here demonstrate that K339 is a key determinant of substrate binding, substrate orientation, and active-site ionization in *Pa*D2HGDH. The evidence supporting these conclusions is presented below.

### K339 Is Important for Substrate d-Malate Binding in *Pa*D2HGDH

Evidence to support this conclusion comes from comparing the reductive half-reaction with d-malate for the K339 variants and the wild-type enzyme at pH 7.4, where *K*_d_ is pH-independent.^19^ pH-independence is a required condition to avoid the contribution from pH effects and to isolate the effect of K339 substitution on substrate binding. Substitution of K339 with methionine weakened substrate binding by ∼5 kJ/mol (ΔΔ*G* is calculated using Footnote[Fn fn1]), consistent with the loss of electrostatic interaction between the lysine NH_3_^+^ group and the substrate C5 carboxylate. The K339Q variant showed a smaller effect of ∼2 kJ/mol, likely due to partial compensation by hydrogen bonding from the glutamine side chain. The K339R variant also showed weaker binding, with a ΔΔ*G* of ∼3 kJ/mol, consistent with the less localized positive charge of the guanidinium group compared with the lysine amino group. Similarly, substitution of K339 with M, Q, or R decreased the energy driving substrate capture that procedes catalysis, as reflected in *k*_cat_/*K*_m_, by 10–15 kJ/mol, further supporting the importance of electrostatic interactions in substrate binding and catalysis by *Pa*D2HGDH. Previous studies similarly show that conserved charged residues play key roles in substrate binding through electrostatic complementarity. In *Saccharomyces cerevisiae* flavin-containing monooxygenase, substitution of K227 with alanine weakened NADPH binding by ∼7 kJ/mol,^37^ while substitution of K238 in *Treponema denticola* cystathionine lyase decreased glycine binding by ∼10 kJ/mol.^38^ Likewise, replacement of E312 with glutamine in choline oxidase reduced choline binding by ∼15 kJ/mol,^39^ and replacement of E87 in *P. aeruginosa* d-arginine dehydrogenase was shown to be essential for recognition of the cationic d-arginine substrate.^40^ Taken together, these findings reinforce the notion that electrostatic interactions mediated by conserved charged residues are a general mechanism for ensuring proper substrate binding across diverse enzyme systems, consistent with our observations for K339 in *Pa*D2HGDH, which helps recognize and position the charged carboxylate moieties of d-malate despite their distance from the site of bond cleavage.

### K339 Is Important for the Optimal Orientation of Substrates for Flavin Reduction in *Pa*D2HGDH

Evidence for this conclusion comes from comparing the reductive half-reaction of K339 variants with that of the wild-type enzyme using d-malate at pH 9.5, where *k*_red_ is pH-independent.^19^ pH independence is a required condition to avoid the contribution from pH effect and to isolate the effect of K339 substitution on the rate of flavin reduction. The *k*_red_ value was 75-fold lower in K339Q (ΔΔ*G* ∼ 11 kJ/mol) and up to 18-fold lower in K339R and K339M (ΔΔ*G* ∼ 7 kJ/mol) than the wild-type enzyme, indicating that the mutation of K339 disrupts the precise electrostatic and hydrogen-bonding network necessary for optimal flavin reduction. The unexpectedly large decrease in *k*_red_ for K339Q suggests that the role of K339 is not purely electrostatic. Loss of the positive charge, together with changes in side-chain length, geometry, and hydrogen-bonding interactions, may favor a less productive substrate orientation, whereas methionine may better preserve the steric reach of lysine despite lacking its positive charge. The presence of positively charged K339 is therefore important for anchoring and properly orienting the substrate C1 carboxylate and C2 hydroxyl groups for hydride transfer to the flavin N5 atom. Consistent with this interpretation, the rate of catalytic turnover, *k*_cat_, also decreased significantly across all variants, supporting the importance of K339 in maintaining the substrate and product orientation and geometry required for efficient catalysis by *Pa*D2HGDH. A previous study was reported in choline oxidase, where substitution of E312 with aspartate decreased the rate of hydride transfer to flavin N5 by 260-fold (ΔΔ*G* ∼ 13 kJ/mol) due to an increased donor–acceptor distance.^39,41^ Similarly, the K339 variants of *Pa*D2HGDH demonstrate that subtle changes in active-site geometry can profoundly alter flavin reduction and the enzyme’s catalytic pathway.

### K339 Maintains the Electrostatic Environment Required for Favorable Substrate C2 Hydroxyl Deprotonation

This conclusion is supported by pH-dependent *K*_d_ measurements of the K339M, determined using a stopped-flow spectrophotometer and compared with previously published data for the wild-type enzyme.^19^ The apparent p*K*_a_ value of the *K*_d_ pH profile for the K339 M variant was ∼10.4, which was ∼1.5 units higher than that of the wild type (p*K*_a_ ∼ 8.9).^19^ A previous study proposed that Zn^2+^ polarizes the active-site water molecule, forming a Zn^2+^–hydrate complex and lowering the p*K*_a_ of water from 14 to ∼9.^17,21^ By analogy, the wild-type *K*_d_ pH profile p*K*_a_ of ∼8.9 observed here likely reflects the formation of a Zn^2+^–OH­(C2) substrate complex. The shift in p*K*_a_ indicates that replacing the positively charged lysine with a nonpolar methionine perturbs the local ionization environment around the Zn^2+^–OH­(C2) substrate complex. In the wild-type enzyme, the negative charge of the C5 carboxylate of an α-hydroxy acid substrate is neutralized by the positive charge of the side chain of lysine, which is likely favorable for the deprotonation of the substrate’s C2-OH. Thus, the p*K*_a_ of the Zn^2+^–OH­(C2) substrate complex is ∼8.9. In K339M, the environment becomes less favorable for the deprotonation of the substrate’s C2-OH at lower pH, due to the presence of a negative charge on the substrate C5 carboxylate. As a result, deprotonation of the C2-OH substrate in K339M requires a higher pH, thereby increasing the apparent p*K*_a_ of the Zn^2+^–OH­(C2) substrate complex.

Notably, the pH profiles of *k*_red_ for the K339M variant and the wild-type enzyme yielded similar p*K*_a_ values of ∼8.3. This p*K*_a_ was previously assigned to H421, which was proposed to act as the proton acceptor in forming the substrate alkoxide required for hydride transfer to the flavin.^19^ A similar increase in histidine basicity has been reported in myoglobin, where H36 exhibits a p*K*_a_ of ∼8 due to a salt link with the carboxylate of E36.^42,43^ Likewise, the p*K*_a_ of H421 is increased from its typical value of ∼6 to ∼8, likely through a histidine–carboxylate interaction^44^ with a nearby D341 residue^15^ and that substitution of K339 does not perturb the ionization environment of the His–Asp dyad.

### Substitution of K339 Perturbs a Solvent-Sensitive Internal Isomerization in the Catalytic Cycle of *Pa*D2HGDH

Evidence for this conclusion comes from the inverse hyperbolic patterns observed in plots of the normalized *k*_cat_ and *k*_cat_/*K*_m_ values for K339Q, and the *k*_cat_ value for K339M, as a function of increasing relative solvent viscosity with both glucose and glycerol. Diffusional processes that result in the generation of the ES complex and decay of the EP complex are indeed ruled out because of the absence of linear viscosity dependence.^36^ The inverse hyperbolic pattern, instead, is symptomatic of an internal isomerization of an enzyme complex that is perturbed by the presence of viscosigen.^36^ For K339Q, the *k*_cat_ and *k*_cat_/*K*_m_ values showed inverse hyperbolic KSVEs, indicating the presence of internal isomerization in the ES complex competent for the flavin reduction, and an isomerization on the EP complex can be ruled out (Scheme 2). In contrast, the overall turnover of the wild-type enzyme is limited by both flavin reduction and product release, and the ES complex isomerization is masked by the slower product-release step, as indicated by a positive slope in the linear solvent viscosity plot.^18^

**Scheme 2:**
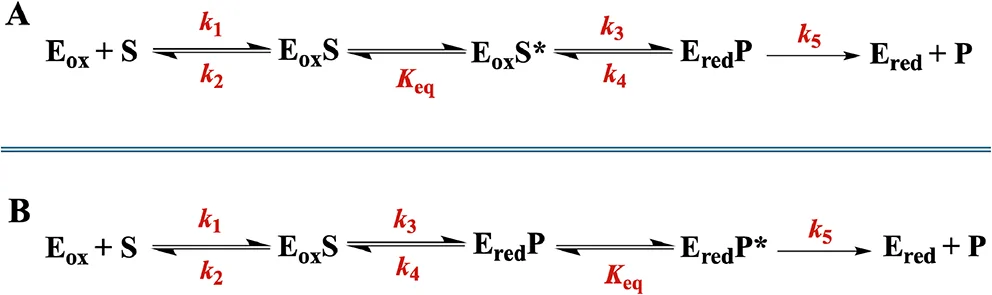
Minimal Kinetic Mechanism for Reductive Half-Reaction of the K339 Variant Enzymes, with Internal Isomerization of the ES Complex in K339Q and K339R (A) and the EP Complex in K339M (B)

Alternatively, the K339R variant showed no effect of solvent viscosity on *k*_cat_ or *k*_cat_/*K*_m_, indicating that diffusional steps, such as substrate dissociation and product release, are not rate-limiting. Because the *k*_cat_ value of 1.02 s^–1^ was still ∼3-fold lower than the 3.4 s^–1^ of *k*_red_, overall turnover cannot be limited solely by hydride transfer. Instead, the data support partial rate limitation of overall catalytic turnover by both hydride transfer and an internal isomerization of the ES complex (Scheme 2), with the latter estimated to occur at 2.3 ± 0.24 s^–1^ using eq 1. Similar viscosity-sensitive internal equilibria have been reported for the E312D variant of choline oxidase with choline as a substrate,^39^ nitronate monooxygenase from *Neurospora crassa* with 3-nitropropionic acid as a substrate,^35^ NAD­(P)­H:quinone oxidoreductases from *P. aeruginosa* with benzoquinone or juglone as a substrate,^34^ the M62A variant and wild-type choline oxidase from *Arthrobacter globiformis* with oxygen as a substrate,^45^ and flavocytochrome b_2_.^46^

8 $$k_{\mathrm{cat}}=\frac{k_{\mathrm{red}}k_{x\prime }}{k_{\mathrm{red}}{+k}_{\mathrm{rev}}{+k}_{x\prime }}$$

## Conclusions

The study demonstrates that K339 plays a crucial role in the substrate binding and catalysis of *P. aeruginosa* d-2-hydroxyglutarate dehydrogenase. Substitution of this conserved active-site lysine with methionine, glutamine, or arginine weakened d-malate binding; decreased *k*_red_, *k*_cat_, and *k*_cat_/*K*_m_; and perturbed the ionization environment of the Zn^2+^–OH­(C2) substrate complex in *Pa*D2HGDH. These results show that K339 provides the electrostatic interactions required for productive substrate binding and for the formation of the catalytically competent ES complex for flavin reduction. Solvent viscosity effects further indicate that K339 modulates conformational equilibria during turnover, thereby influencing internal isomerization steps in ES and EP complexes. Together, these findings support a model in which the conserved lysine residue at the active-site entrance anchors the substrate tail, properly orients the C1 carboxylate and C2 hydroxyl for catalysis, and fine-tunes the internal isomerization of the catalytic cycle in *Pa*D2HGDH. Given the essential role of *Pa*D2HGDH in *P. aeruginosa* metabolism, the mechanistic insights gained here provide a framework for engineering D2HGDH homologues and for designing inhibitors that exploit the electrostatic and dynamic contributions of this conserved positive charge residue. Future structural and stability studies of the K339 variants may further clarify how substitutions at this position affect the enzyme structure and function.

## Experimental Procedure

### Materials

The restriction enzyme DpnI was obtained from New England Biolabs (Ipswich, MA), while *Pfu* DNA polymerase was purchased from Agilent Technologies (Alpharetta, GA) and oligonucleotides were from Sigma Genosys (The Woodlands, TX). *E. coli* Rosetta­(DE3)­pLysS cells and the pET20b­(+) expression vector were supplied by Novagen (Madison, WI). The DH5α *E. coli* strain was obtained from Life Technologies, Inc., and the QIAprep spin miniprep, QIAquick PCR purification, and QIAquick gel extraction kits were purchased from Qiagen (Valencia, CA). The pET20b­(+) plasmid containing the *P. aeruginosa* D2-hydroxyglutarate dehydrogenase (*Pa*D2HGDH) gene with a C-terminal 6× His tag was designed in-house and synthesized by GenScript (Piscataway, NJ). BSA was obtained from Promega (Madison, WI). Luria–Bertani agar and broth, chloramphenicol, IPTG, lysozyme, sodium hydrosulfite (dithionite), phenazine methosulfate (PMS), PMSF, and zinc chloride were purchased from Sigma-Aldrich (St. Louis, MO). Ampicillin was obtained from ICN Biomedicals (Aurora, OH), d-2-hydroxyglutarate was from Millipore Sigma (Burlington, MA), and d-malate was from Alfa Aesar (Haverhill, MA). Glucose, glycerol, and all other commercially available reagents were of high purity.

### Site-Directed Mutagenesis, Protein Expression, and Protein Purification of K339 Variants

The genes encoding the *Pa*D2HGDH K339 variants were generated using a pET20b­(+) plasmid containing the wild-type PA0317 gene as the template together with mutagenic primers carrying the corresponding substitutions. Amplification with *Pfu* DNA polymerase was verified by agarose gel electrophoresis. The PCR products were purified with the QIAquick PCR Purification Kit and treated with DpnI at 37 °C for 2 h. The resulting DNA was used to transform DH5α *E. coli* cells.

The plasmids pET20b­(+)/PA0317/K339M, pET20b­(+)/PA0317/K339Q, and pET20b­(+)/PA0317/K339R were subsequently sent to Psomagen, Inc. for sequencing to verify the desired mutations. Constructs containing the correct substitutions were introduced into chemically competent *E. coli* Rosetta­(DE3)­pLysS cells by heat shock for protein expression, and the resulting cells were stored at −80 °C. Expression and purification of the K339 variants were performed using the previously described protocol for the wild-type enzyme.^17,18^ The concentration of the purified total enzyme was measured using the Bradford method.^33^

### UV–Visible Absorption Spectroscopy and Extinction Coefficient Determination

An Agilent Technologies HP 8453 PC diode-array spectrophotometer, thermostated with a water bath, was used to measure the UV–visible absorbance of enzyme-bound flavin in 25 mM NaPO_4_, 10% glycerol, and 1 mM ZnCl_2_, pH 7.4, at 25 °C. The spectroscopic properties of purified wild-type *Pa*D2HGDH and the K339 variants were evaluated by recording absorption spectra from 280 to 800 nm in a quartz cuvette with a 1 cm path length. The resulting data were analyzed using KaleidaGraph software (Synergy Software, Reading, PA). The extinction coefficients of the purified wild-type and variant enzymes were determined after extraction of the FAD cofactor by heat denaturation.^47,48^ Before denaturation, the enzymes were passed through a PD-10 desalting column and subsequently heated at 100 °C for 20, 30, and 40 min.^49,50^ The precipitated protein was removed by centrifugation at 20,000*g*. The concentration of released FAD was measured spectroscopically using an ε_450_ of 11,300 M^–1^ cm^–1^ for free FAD.^48,50^ The concentration of the FAD-bound enzyme was calculated from the absorbance at 450 nm using the experimentally determined ε_450_ of 12,500 M^–1^ cm^–1^ for enzyme-bound FAD.

### Reductive Half-Reaction of K339 Variants

The anaerobic reduction of the *Pa*D2HGDH K339 variants with d-malate was monitored at pH 7.4 and 9.5 using an SF-61DX2 Hi-Tech KinetAssyst high-performance stopped-flow spectrophotometer (Bradford-on-Avon, U.K.), maintained at 25 °C with a water bath. The experiments were performed using the same procedures and conditions previously established for the wild-type enzyme.^18,19^ The instrument, buffers, substrates, and enzyme solutions were made anaerobic according to the standard procedure.^18,19^ After mixing, the enzyme concentration was ∼10 μM, while d-malate concentrations ranged from 10 to 250 mM for K339M, 2–160 mM for K339Q, and 2–225 mM for K339R to maintain pseudo-first-order conditions.

pH dependence of the K339M reductive half-reaction was examined from pH 7.0 to 10.5 in 25 mM Good’s buffer (0.1 M ACES, 0.052 M Tris, and 0.052 M ethanolamine) containing 1 mM ZnCl_2_ at 25 °C, under conditions previously established for the wild-type enzyme.^19^ Following mixing with the substrate, the enzyme concentration was ∼10 μM and d-malate concentrations ranged from 10 to 180 mM. Flavin reduction was followed by monitoring the decrease in absorbance at 450 nm upon mixing the enzyme with d-malate. Absorbance changes were measured in triplicate at each substrate concentration, and the mean values were reported. Replicate measurements typically varied by ≤5%.

### Steady-State Kinetics of K339 Variants

The steady-state kinetic parameters of the *Pa*D2HGDH K339 variants with the physiological substrate D2HG or the alternative substrate d-malate were determined from initial rates of PMS-dependent oxygen consumption using a Clark-type oxygen electrode (Hansatech Instruments). PMS served as the electron acceptor because d-2-hydroxyglutarate dehydrogenase is a true dehydrogenase and does not react directly with molecular oxygen. Oxygen consumption therefore reflects enzymatic activity through the spontaneous reaction of reduced PMS with O_2_. Assays were performed in 25 mM NaPO_4_, pH 7.4, at 25 °C with a fixed saturating PMS concentration of 2 mM. The saturating PMS concentration was established by measuring variant enzyme activity at 1, 2, and 3 mM PMS in the presence of 60 mM d-malate. d-malate and D2HG were varied over concentration ranges of 0.2–60 and 0.02–1 mM, respectively. The concentrations of K339M, K339Q, and K339R were 4.6, 2.4, and 0.7 μM, respectively.

### Kinetic Solvent Viscosity Effects of K339 Variants

The effect of relative solvent viscosity on the kinetic parameters *k*_cat_ and *k*_cat_/*K*_m_ of the K339 variants was examined at 25 °C in 25 mM NaPO_4_, pH 7.4, using glucose or glycerol as viscosigens under the same conditions previously established for the wild-type enzyme.^18^ The viscosities of reaction mixtures containing varying amounts of glucose (0–34%, m/m, η_rel_ = 1.0–3.6 cP) or glycerol (0–40%, m/m, η_rel_ = 1.0–3.5 cP) were estimated at 25 °C using relative viscosity data reported at 20 °C by Lide.^51^

### Data Analysis

Initial rates of oxygen consumption were recorded using an Oxy-32 computer-connected oxygen-monitoring system (Hansatech Instruments Ltd., Norfolk, U.K.). Steady-state kinetic parameters were determined by fitting the experimental data to the Michaelis–Menten equation (eq 2) with KaleidaGraph software (Synergy Software, Reading, PA).

1 $$v_{o}/e=\frac{k_{\mathrm{cat}}\left[S\right]}{K_{m}+\left[S\right]}$$

where *v*_o_ represents the initial velocity, *e* is the enzyme concentration, and *k*_cat_ represents the turnover rate at saturating concentrations of both substrates.

Stopped-flow traces collected using KinetAsyst 3 (TgK-Scientific, Bradford-on-Avon, U.K.) software were analyzed according to the double-exponential function given in eq 2.

2 $$A=B_{1}{e_{obs1^{t}}}^{-k}+B_{2}e^{-k}obs2^{t}+C$$

In eq 2, *A* is the absorbance at 450 nm at time *t*, *B*_1_ and *B*_2_ are the amplitudes associated with the absorbance changes, *k*_obs1_ and *k*_obs2_ are the corresponding observed rate constants, and *C* represents the offset accounting for the residual absorbance of the enzyme-bound reduced flavin at an infinite time.

The dependence of the observed flavin-reduction rate constants on d-malate concentration was analyzed using eq 5. In this equation, *k*_red_ represents the rate constant for flavin reduction at saturating d-malate, *k*_rev_ represents the reverse rate constant describing conversion of the enzyme–product complex (EP) to the enzyme–substrate complex (ES), and *K*_d_ is the apparent dissociation constant for d-malate.

3 $$k_{obs}=\frac{k_{\mathrm{red}}\left[D‐malate\right]}{K_{d}+\left[D‐malate\right]}+k_{rev}$$

The solvent viscosity effects on *k*_cat_ and *k*_cat_/*K*_m_ were analyzed using eqs 4 and 6. Here, (*k*)_o_ and (*k*)_η_ represent the kinetic parameter measured in the absence and presence of viscosigen, respectively. *S* represents the magnitude of the viscosity dependence, η_rel_ is the relative viscosity of the buffered solution, and *A* and *B* are parameters describing the observed inverse-hyperbolic dependence.

4 $$\frac{{\left(k\right)}_{o}}{{\left(k\right)}_{\eta }}=S\left(\eta_{rel}-1\right)+1$$

5 $$\frac{{\left(k\right)}_{o}}{(k_{\eta }}=\frac{1}{1+A\left(\frac{\eta_{rel}-1}{\left(\eta_{rel}-1\right)+B}\right)}$$

The pH dependence of *k*_red_ was analyzed using eq 6, where *k*_red_(lim) represents the limiting, pH-independent rate constant for flavin reduction at high pH and p*K*_a_ represents the apparent ionization constant of a group that must be unprotonated for flavin reduction.

6 $$\log\left(k_{red}\right)=\frac{k_{\mathrm{red}}\left(\lim\right)}{1+\frac{10^{-\mathrm{pH}}}{10^{{-pK}_{a}}}}$$

The pH dependence of *K*_d_ was analyzed using eq 7, which describes a profile with a slope of +1 at high pH and a low-pH plateau corresponding to the limiting, pH-independent *K*_d_.

7 $$\log\left(K_{d}\right)=\log\left[K_{d}\left(\lim\right)\cdot \left(1+10^{pH-{pK}_{a}}\right)\right]$$

## Supplementary Material



## Data Availability

All data are contained within the manuscript, except that reported in the Supporting Information.

## Footnotes

1 ΔΔ*G*° = *RT* ln­(*K*_d_,_variant_/*K*_d_,_WT)_, with *R* = 8.314 J mol^–1^ K^–1^ and *T* = 298.1 K.

## Notes

Anaerobic reduction of K339Q and K339R variants with d-malate at pH 7.4, SDS-PAGE analysis of wild-type *Pa*D2HGDH and K339 variants, and effect of pH on the rapid-reaction kinetic parameters of K339 M with d-malate (PDF)

## ⊥

Department of Chemistry, Princeton University, Frick Chemistry Laboratory, Corner of Washington Road and Scholar Way, Princeton, New Jersey 08544, United States

## Abbreviations

*Pa*D2HGDH: *P. aeruginosa* d-2-hydroxyglutarate dehydrogenase
D2HG: d-2-hydroxyglutarate
KSVE: kinetic solvent viscosity effect
ES complex: enzyme–substrate complex
EP complex: enzyme–product complex
PP loop: pyrophosphate-binding loop
FAD: flavin adenine dinucleotide
