# Supplementary material for: Electrostatic Contribution of K339 to the Flavin Reduction of Pseudomonas aeruginosa PAO1 d‑2-Hydroxyglutarate Dehydrogenase
Source: ACS Omega. 2026 Oct 21;11(43):67792–804. doi: 10.1021/acsomega.6c09466 (PMC13736859; doi:10.1021/acsomega.6c09466)
Supplement: Supplementary file 1 [file ao6c09466_si_001.pdf]

# *Supporting Information*: Electrostatic contribution of K339 to the flavin reduction of *Pseudomonas aeruginosa* PAO1 D-2-hydroxyglutarate dehydrogenase

Bilkis Mehrin Moni[1,4], Joanna Afokai Quaye[1#], and Giovanni Gadda[1,2,3,*]

[1]Departments of Chemistry, [2]Biology, and [3]The Center for Diagnostics and Therapeutics, [4]Molecular Basis of Disease, Georgia State University, Atlanta, GA 30302-3965

**Corresponding author**

*To whom correspondence should be addressed: Giovanni Gadda, Department of Chemistry, Georgia State University, P.O. Box 3965, Atlanta, GA 30302-3965, U.S.A.

Phone: (404) 413-5537

FAX: (404) 413-5505

EMAIL: ggadda@gsu.edu

**Author**

#Joanna Afokai Quaye, current address differs from the research affiliation, Department of Chemistry, Princeton University, Frick Chemistry Laboratory, Corner of Washington Road and Scholar Way, Princeton, 08544, New Jersey, U.S.A.

**Keywords**

D-2-hydroxyglutarate dehydrogenase, *Pseudomonas aeruginosa,* substrate binding, catalysis, active-site ionization, flavin reduction, electrostatic, isomerization.

## Supporting Information

### Supporting Figure 1

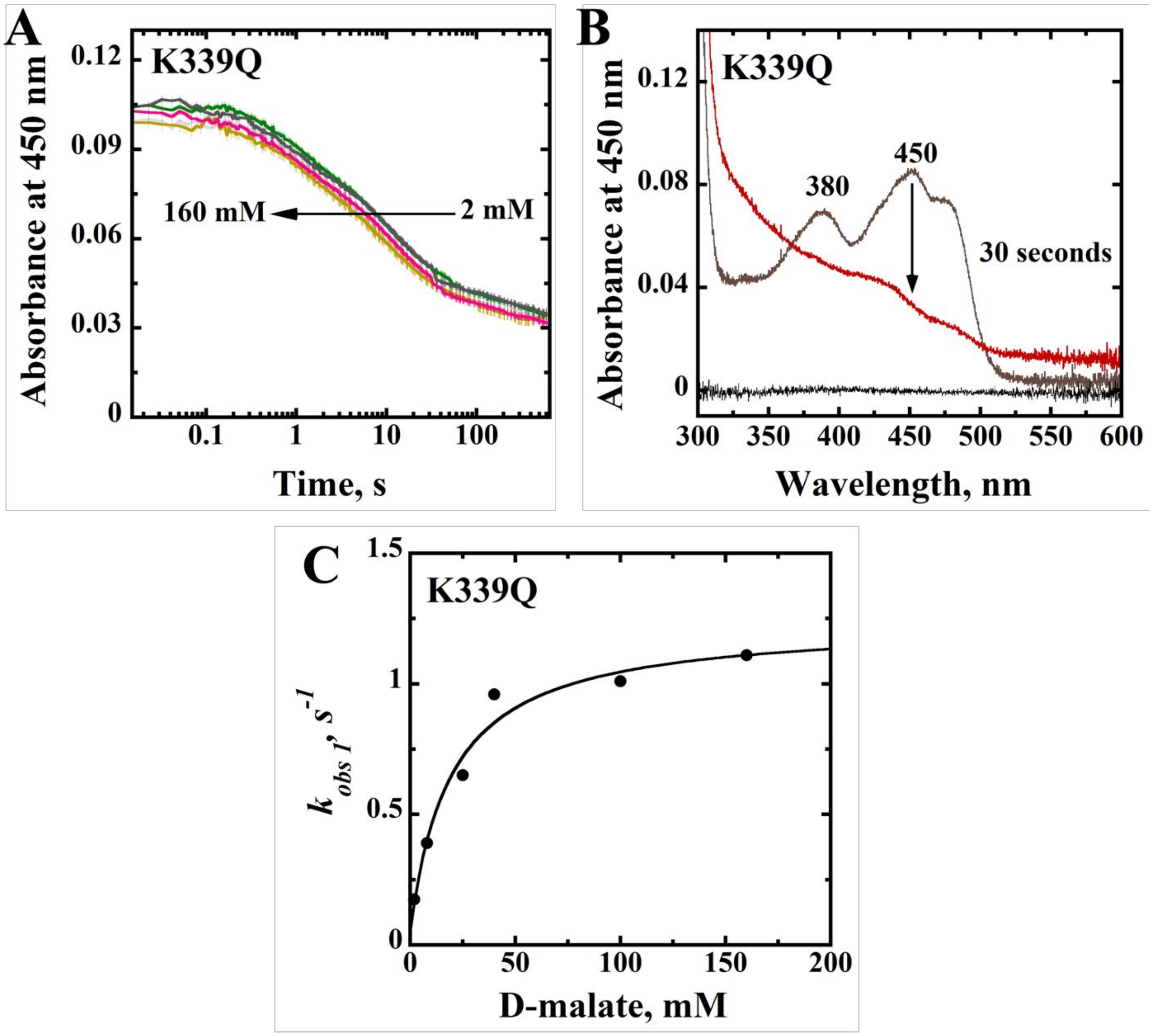


**Figure S1: Anaerobic reduction of K339Q** variant **with D-malate at pH 7.4.** A, stopped-flow traces of K339Q at 450 nm with varying concentrations of D-malate (2-160 mM) fit the **eq 2**. Note the log time scale. For clarity, one out of every 10 experimental points is shown (vertical lines). The instrument dead time is 2.2 ms. B, absorption spectra of K339Q showing the fully oxidized flavin before reduction (gray trace) and fully reduced flavin after reduction with 100 mM of D-malate (red trace). C, concentration dependence of the observed rate constant ($k_{obs1}$) for flavin reduction of K339Q with D-malate, fit to the **eq 3**. The experiment was carried out in 25 mM $NaPO_4$ using an SF-61DX2 Hi-Tech KinetAsyst high-performance stopped-flow spectrophotometer, thermostated at 25°C, and equipped with a photomultiplier tube detector under anaerobic conditions.

**Supporting Figure 2**

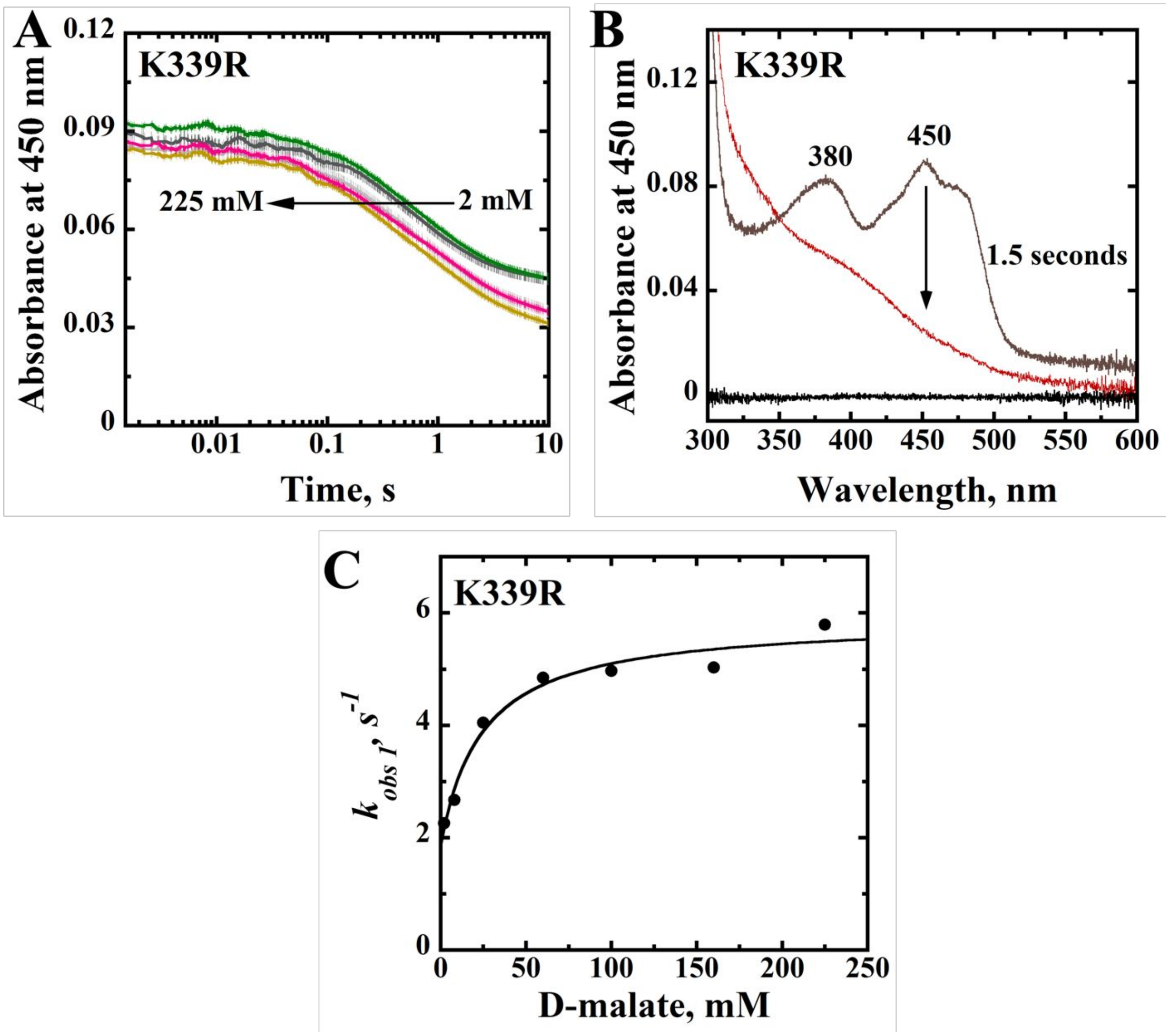


**Figure S2: Anaerobic reduction of K339R** variant **with D-malate at pH 7.4.** A, stopped-flow traces of K339R at 450 nm with varying concentrations of D-malate (2-225 mM) fit the **eq 2**. Note the log time scale. For clarity, one out of every 10 experimental points is shown (vertical lines). The instrument dead time is 2.2 ms. B, absorption spectra of K339R showing the fully oxidized flavin before reduction (gray trace) and fully reduced flavin after reduction with 100 mM of D-malate (red trace). C, concentration dependence of the observed rate constant ($k_{obs1}$) for flavin reduction of K339R with D-malate, fit to the **eq 3**. The experiment was carried out in 25 mM $NaPO_4$ using an SF-61DX2 Hi-Tech KinetAsyst high-performance stopped-flow spectrophotometer, thermostated at 25°C, and equipped with a photomultiplier tube detector under anaerobic conditions.

Supporting Figure 3

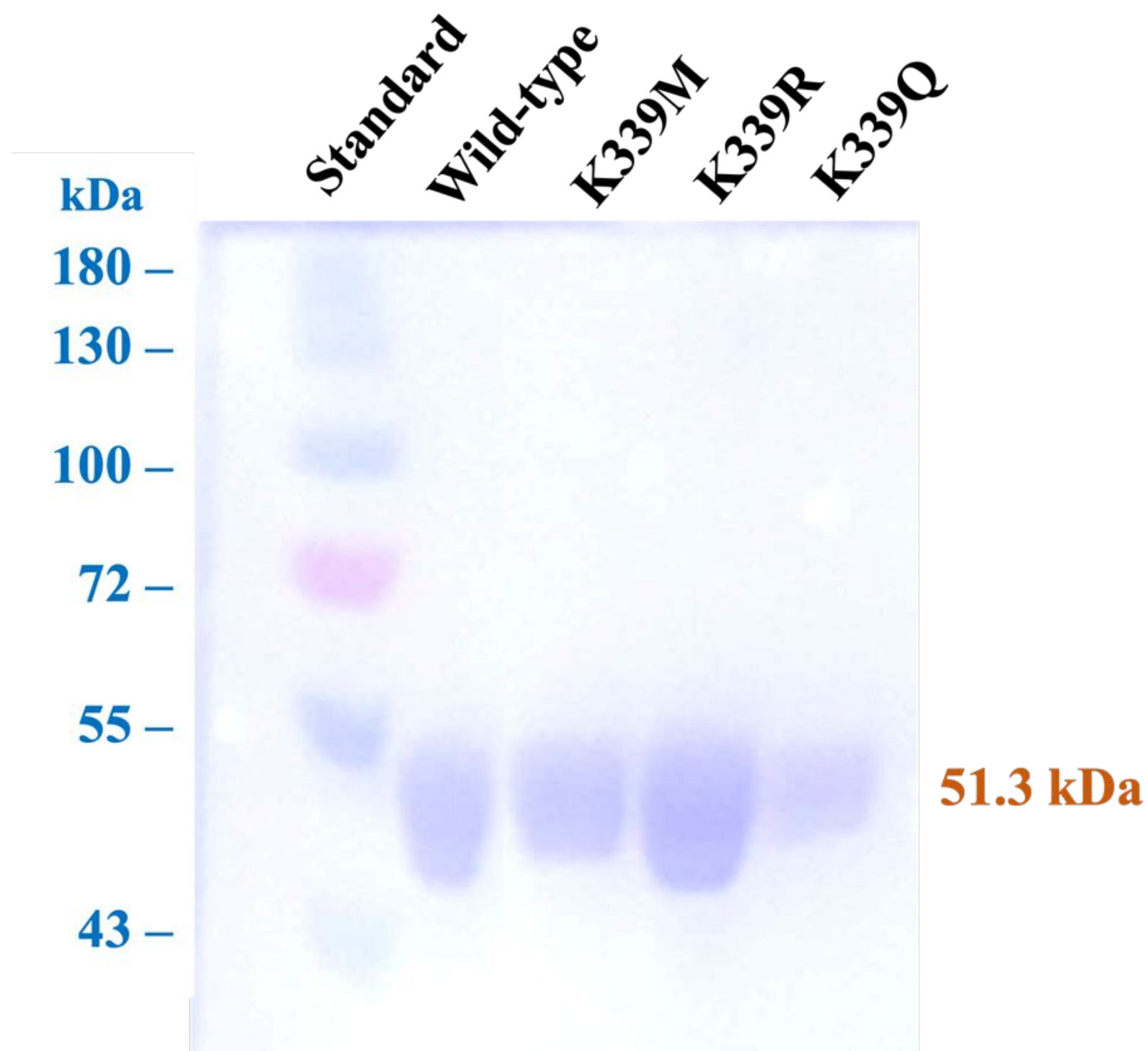


**Figure S3. SDS-PAGE analysis of wild-type D-2-hydroxyglutarate dehydrogenase and K339 variants.** SDS-PAGE evaluation of the purified recombinant proteins revealed a predominant single band at approximately 51.3 kDa, indicating a high level of protein purity.

**Supporting Table 1**

**Table S1: *Effect of pH on the rapid-reaction kinetic parameters of K339M with D-malate**

| pH | $k_{red}$, $s^{-1}$ | $K_d$, mM |
|---|---|---|
| 10.5 | 255 ± 21 | 161 ± 23 |
| 10.0 | 234 ± 14 | 114 ± 14 |
| 9.5 | 225 ± 22 | 102 ± 20 |
| 9.0 | 212 ± 15 | 90 ± 13 |
| 8.5 | 147 ± 9 | 74 ± 10 |
| 8.0 | 74 ± 4 | 77 ± 10 |
| 7.5 | 32 ± 1 | 65 ± 5 |
| 7.0 | 19 ± 1 | 60 ± 10 |

*Effect of pH on the rapid-reaction kinetic parameters, $k_{red}$ and $K_d$ of K339M with D-malate. Each value is shown with the associated standard error from fitting the kinetic data with **eq 3**. Assays were carried out in Good's buffer (0.1 M ACES, 0.052 M Tris, 0.052 M ethanolamine), and 1 mM $ZnCl_2$ at 25 $^{o}$C. D-malate varied from 10 mM to 200 mM, with enzyme concentration ranging from 15.8 μM to 16.8 μM to maintain pseudo-first-order conditions. Standard errors are from individual fits of the kinetic data.
